# Choosing the Optimal Rat Stock as a Model for Research into Pharmacological Correction of Male Sexual Dysfunction

**DOI:** 10.17691/stm2021.13.6.04

**Published:** 2021-12-28

**Authors:** Z.D. Zhuravleva, N.A. Titova, I.V. Mukhina, M.Ya. Druzin

**Affiliations:** Assistant, Department of Physiology and Anatomy, Institute of Biology and Biomedicine; National Research Lobachevsky State University of Nizhni Novgorod, 23 Prospekt Gagarina, Nizhny Novgorod, 603950, Russia;; PhD Student, Department of Neurotechnologies; National Research Lobachevsky State University of Nizhni Novgorod, 23 Prospekt Gagarina, Nizhny Novgorod, 603950, Russia;; Professor, Director of the Institute of Fundamental Medicine; Privolzhsky Research Medical University, 10/1 Minin and Pozharsky Square, Nizhny Novgorod, 603005, Russia; Head of the Department of Normal Physiology named after N.Y. Belenkov; Privolzhsky Research Medical University, 10/1 Minin and Pozharsky Square, Nizhny Novgorod, 603005, Russia;; Docent, Department of Integrative Medical Biology; Umeå Universitet, 901 87, Umea, Sweden

**Keywords:** sexual behavior, social activity, anxiety, glycine receptors, medial preoptic area

## Abstract

**The aim of the study** is to identify the mechanisms mediating differences in sexual behavior between Sprague Dawley and Wistar rats, in order to choose the optimal stock for research into pharmacological correction of male sexual dysfunction.

**Materials and Methods:**

The experiments were carried out on sexually mature male rats of two stocks (Sprague Dawley and Wistar) weighing 350–450 g and aged 3 to 6 months. The comparative study of animal behavior was performed using standard tests for social interaction, locomotor activity, and anxiety level, as well as male mating behavior patterns. In order to determine the role of hypothalamic glycine receptors in the male sexual behavior, pharmacological manipulations of glycine receptor activity during mating with receptive females were conducted via bilateral intracerebral microcannulas implanted in the medial preoptic area (mPOA) of the male rat anterior hypothalamus.

**Results:**

The obtained results revealed statistically significant inter-stock differences in sexual behavior at the final consummatory stage of both intact animals and those after pharmacological activation of glycine receptors in the mPOA. The number of anxiety-related grooming patterns in the Open Field test significantly differed between the stocks for both intact animals and those after pharmacological activation of glycine receptors; the observed differences disappeared after the mPOA glycine receptors were blocked. In the Crowley test of social interaction, no significant difference was found between the stocks.

Thus, the revealed difference in sexual behavior between Sprague Dawley and Wistar male rats is likely due to the difference in the level of anxiety, which, in turn, may be associated with difference in the mechanisms of glycinergic neurotransmission in the hypothalamic mPOAs of these rats.

**Conclusion:**

To study the relationship between the level of anxiety and sexual behavior, the choice of the Wistar rat stock is optimal since the male sexual behavior in this stock is more sensitive to stress than that in Sprague Dawley rats. However, to model male sexual dysfunction not associated with anxiety, the use of Sprague Dawley male rats should be preferred as these animals show more stable sexual behavior, which is less dependent on the level of anxiety.

## Introduction

An important part of both fundamental and applied preclinical research is the experimentation on animal models; among those, laboratory rodents, in particular, inbred lines and outbred stocks of mice and rats are used most often [1–3]. Various aspects of sexual behavior are usually studied using the Sprague Dawley and Wistar rat outbred stocks. Despite its importance for selection of the optimal animal model, little is known about the difference in sexual behavior between rats of these two stocks [4, 5].

Recently, our research group has reported a significant difference between Sprague Dawley and Wistar rats in the sexual behavior of intact males and males after pharmacological modulation of glycine receptor activity in the medial preoptic area (mPOA) [6, 7]. The mPOA involvement in control of both social behavior and anxiety [8, 9] is likely one of the main factors determining the observed difference as sexual behavior includes social and emotional components as well. Being an important part of the brain neural circuits, which integrate homeostatic information the mPOA may also link reproductive behavior [10] with a number of stress-related disorders of cardiovascular and metabolic systems [11]. The mPOA is characterized by a high level of glycine receptors [12]. Their dysfunction was reported to contribute to severe neurological disorders, including hyperekplexia and epilepsy [13]. Given the functional role of mPOA, the glycine receptors expressed in mPOA could be a potential therapeutic target for the treatment of disorders associated with male sexual behavior.

**The aim of the study** is to identify the mechanisms mediating difference in sexual behavior between male Sprague Dawley and Wistar rats, in order to choose the optimal stock for research into pharmacological correction of male sexual dysfunction.

## Materials and Methods

The experiments were carried out on sexually mature male and female rats of the Sprague Dawley and Wistar stocks, aged from 3 to 6 months, weighing 350–450 g. Females were used only to test the sexual behavior of males. The study was approved by the Ethics Committee of Umeå University (Sweden). All experimental and surgical procedures were performed in accordance with the standards specified in the Guide for the Care and Use of Laboratory Animals (ILAR publication, 1996, National Academy Press) and the GOST 33216-2014 standard “Rules for the maintenance and care of laboratory rodents and rabbits”.

The process of pre- and postoperative testing was identical for all behavioral techniques used. The standard scheme included a single preoperative test of intact animals followed by bilateral cannula implantation into the mPOA. After a 7-day recovery period, testing was resumed. Animals were randomly injected with a solution of glycine, 1 mM (glycine receptor agonist), strychnine, 20 μM (glycine receptor blocker), and Ringer’s solution for warm-blooded animals as a control. The interval between the tests was 3 days. After the completion of the experiments, histological examination of the brain tissue from the cannulated area was carried out.

### Recording the behavioral parameters during mating

The scheme for testing the sexual behavior of males, the surgical and pharmacological preparation of receptive females for mating, the method for analyzing the parameters of male sexual behavior, as well as the procedure for bilateral intracerebral cannulas implantation, and the method of administration of glycine receptor agonists and antagonists into the mPOA were described in our earlier publications [6, 7]. To assess the efficiency of copulation performance of rats under study, the intromission index was calculated [14].

### Recording the behavioral parameters during social interaction in the Crowley test

The experimental setup included a rectangular box made of transparent plastic, 90×60×34 cm in size, which was divided into 3 compartments of 60×30 cm each (A, B, and MID). The behavioral testing included 3 five-minute stages. With the help of the SMART Video Tracking System v. 3.0 (Panlab, Spain), the time spent by an animal in each of the three compartments was automatically recorded following the traditional Crowley test protocol.

### Recording the behavioral parameters in the open field test

The Open Field (Panlab) experimental setup was a 100×100 cm pad bounded by 50 cm high opaque walls. The field was divided into 20×20 cm squares: 16 outer squares located along the field perimeter and 9 inner squares. During testing, the frequency and duration of grooming were recorded, as well as the pattern of sequential movements during the grooming, which made it possible to observe the phenomenon of “incomplete grooming”.

### Statistical data processing

Statistical analysis of the data obtained during experimentation was carried out using the Prism Windows 5 software (GraphPad, USA). The inter-stock differences were analyzed using the nonparametric Mann–Whitney U-test because the obtained data did not obey the normal distribution pattern as evidenced by the Shapiro–Wilk test. The cut-off value of statistical significance when testing the null hypotheses was taken as 0.05. The results are presented as median and percentiles — Me [25; 75].

## Results

### Intact males

#### Analysis of sexual behavior

Comparing the appetitive phase of sexual behavior by the “intromission latency duration” revealed no significant differences (p=0.38) between the intact Sprague Dawley males (119.0 [44.5; 212.5] s; n=9) and the Wistar males (69.0 [26.0; 173.0] s; n=11). During both the appetitive and consummatory phases, the Wistar male rats mounted rarely, therefore, it was not possible to statistically compare the two stocks using the “mounting latency duration”. The value of this parameter in Sprague Dawley rats was 91.0 [45.5; 120.5] s.

**Figure 1.**
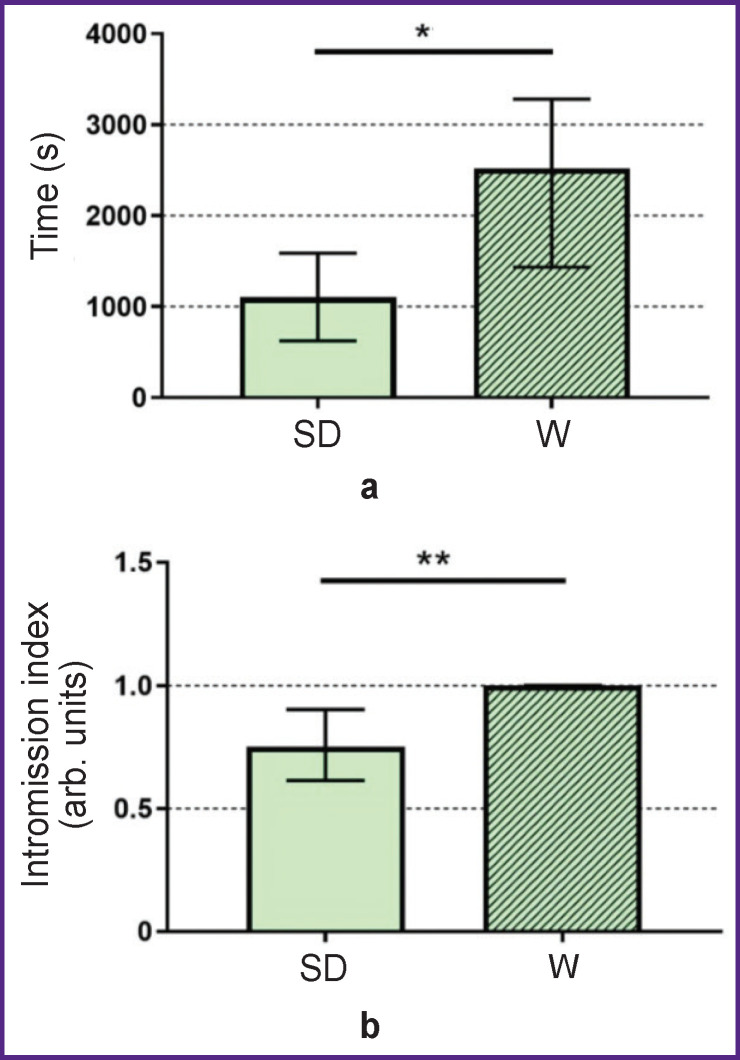
Duration of the ejaculation latency period (a) and the intromissions per session index (b) in intact Wistar (W) and Sprague Dawley (SD) male rats The absolute values are presented; * p<0.05; ** p<0.001

The consummatory phases of sexual behavior in intact males of the Sprague Dawley and Wistar stocks significantly differed (p<0.05) in such parameters as the “ejaculation latency duration” and the “intromissions per session” index. Thus, the “ejaculation latency duration” was twice longer in Wistar male rats (2517.0 [1435.0; 3282.0] s; n=11) compared to Sprague Dawley rats (1102.0 [624.5; 1589.0] s; n=9), p=0.01 (Figure 1 (a)). Since the Wistar stock males almost did not mount, their “intromissions per session” index was higher (1.0 [1.0; 1.0] arb. units; n=11) than that of the Sprague Dawley males (0.75 [0.61; 0.90] arb. units; n=9), p=0.0002 (Figure 1 (b)).

Thus, the experimental results show significant differences in the male sexual behavior between the two stocks. In the Sprague Dawley rats, it took less time to reach ejaculation, which indicated a higher efficiency of copulation performance of this stock compared to the Wistar stock males [15].

#### Analysis of social interaction

At the first stage of testing, in the absence of other animals inside the experimental box, males of both stocks spent about the same time in each of the three compartments (A, B, MID). At the second stage (compartment A — a resident inside, compartment B — vacant), the males of both stocks spent more time in the “resident” compartment. At the third stage (compartment A — a resident, compartment B — an intruder), the males of both stocks spent more time in the “intruder” compartment. This behavior is considered normal [16].

Thus, the similarity of the social activity between the Sprague Dawley and Wistar males in the Crowley test indicates that social behavior is not a factor that determines the differences in the male sexual behavior of these two stocks.

#### Analysis of animal anxiety

**Figure 2.**
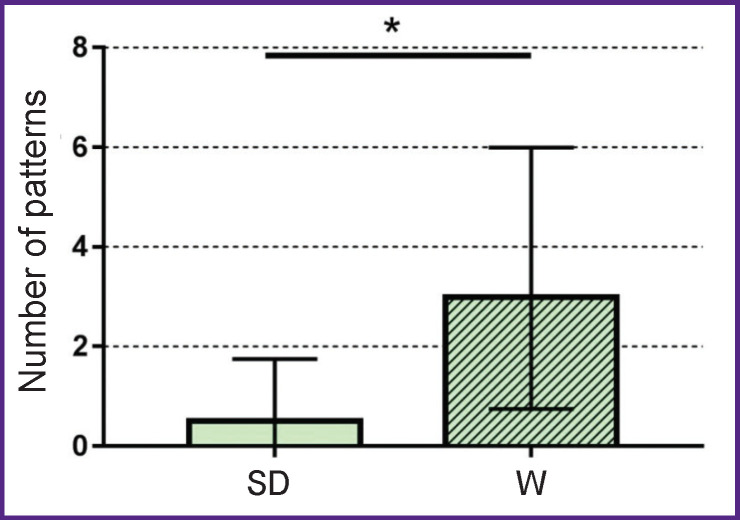
The number of grooming patterns in the outer squares of the Open Field in intact male Wistar (W) and Sprague Dawley (SD) rats The absolute values are presented; * p<0.05

Analysis of the behavioral parameters revealed statistically significant differences in the level of anxiety. Thus, the number of grooming patterns observed in the outer squares of the Open Field was higher in male Wistar rats (3.0 [0.75; 6.0]; n=10) compared to male Sprague Dawley rats (0.5 [0; 1.75]; n=12), p=0.03 (Figure 2). The grooming procedure in Wistar rats was characterized as “incomplete grooming”.

The obtained results indicate that the differences in sexual behavior between males of these two stocks are due to the higher level of anxiety in Wistar rats.

### Males after bilateral microinjections of glycine and strychnine into the medial preoptic area

#### Analysis of sexual behavior

After inhibiting the neural networks with bilateral microinjections of 1 mM glycine, 4 out of 8 male Wistar rats showed either complete (no ejaculation) or partial inhibition of sexual behavior, while Sprague Dawley males retained the ability to copulate in 100% of cases. Moreover, the “ejaculation latency duration” after microinjections of glycine in Sprague Dawley males decreased to 887.5 [556.0; 1063.0] s, which indicated an increased efficiency of copulation performance [15]. Due to the small number of Wistar rats who performed a full-fledged copulation after microinjections of glycine (n=4), statistical comparison between the two stocks was not possible at this stage.

**Figure 3.**
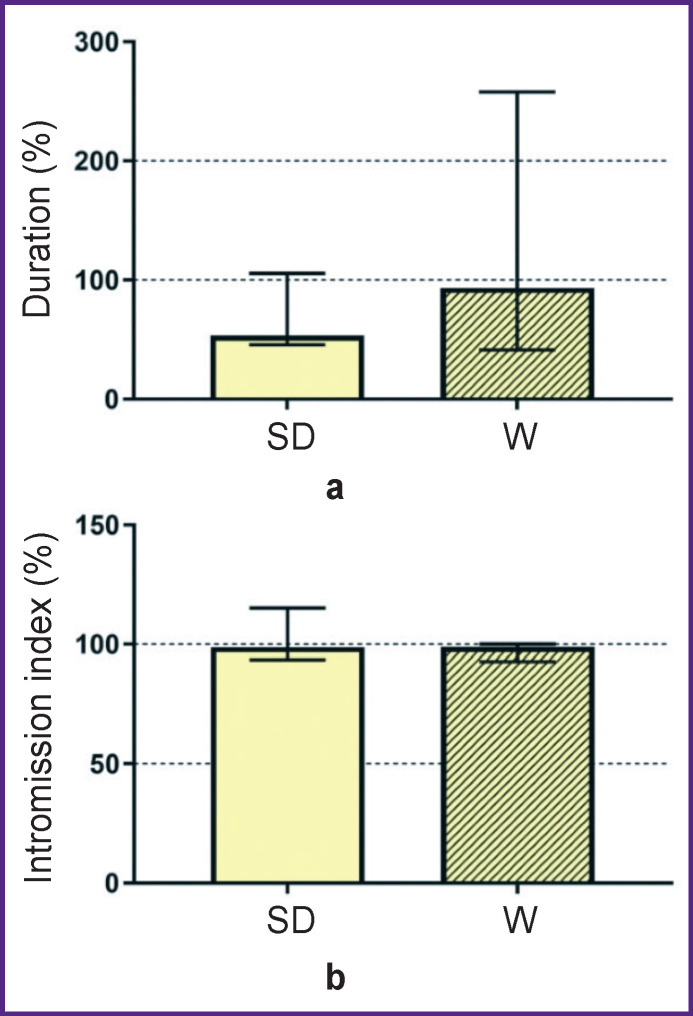
Duration of the ejaculation latency period (a) and the intromissions per session index (b) in male Sprague Dawley (SD) and Wistar (W) rats after bilateral microinjection of strychnine solution (20 μM) in the medial preoptic area The values are normalized per the reference solution

After bilateral microinjection of 20 μM strychnine into the mPOA of male Wistar rats, the ejaculation latency period reduced to 1905.0 [994.5; 3078.0] s per session compared with a longer delay or complete absence of ejaculation during stimulation of inhibition, due to which the statistically significant difference between the two stocks in this parameter disappeared (Figure 3 (a)).

In addition, microinjections of 20 μM strychnine led to the appearance of the “mounting patterns” in Wistar males. As a result, the “intromission index” values became similar between the two stocks (Figure 3 (b)). Along with that, microinjections of strychnine had practically no effect on the “ejaculation latency duration” in Sprague Dawley male rats (1044.0 [536.5; 1497.0] s), which remained close to the baseline values.

Thus, activation of the glycinergic inhibition in the mPOA neural networks led to complete or partial disappearance of sexual behavior in male Wistar rats; the same glycinergic inhibition increased the efficiency of the copulation performance in Sprague Dawley males. The antagonist of glycine receptors strychnine facilitated the restoration of copulation performance in male Wistar rats but it had no effect on sexual behavior of the Sprague Dawley rats.

#### Analysis of social activity

There were no statistically significant differences in social activity (Crowley test) between Sprague Dawley and Wistar stocks after bilateral microinjections of either glycine or strychnine.

#### Analysis of anxiety

**Figure 4.**
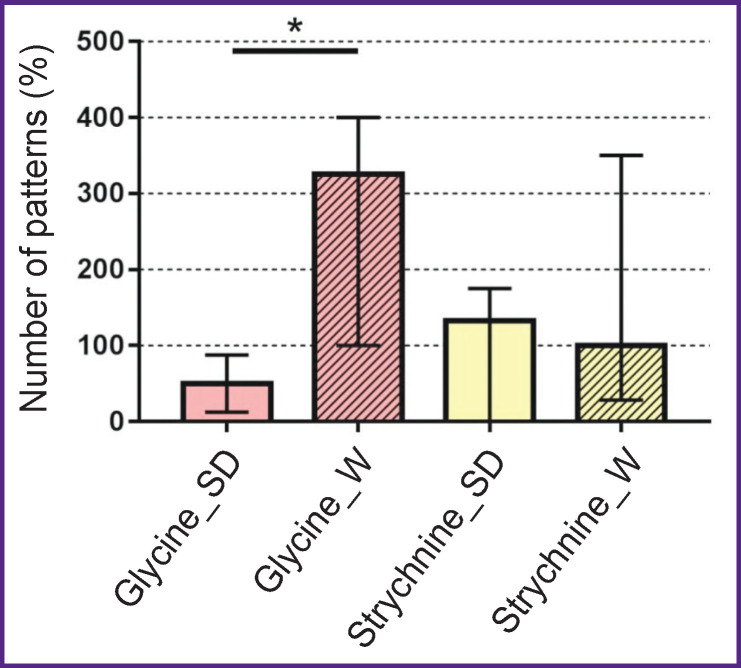
The number of grooming patterns in the outer squares of the Open Field in male rats of Sprague Dawley (SD) and Wistar (W) stocks after bilateral microinjection of glycine (1 mM) and strychnine (20 μM) solutions in the medial preoptic area The values are normalized per the reference solution; * p<0.05

In male Wistar rats, pharmacological intervention into the mPOA glycinergic synaptic transmission by 1 mM glycine increased the number of “incomplete grooming patterns” in the outer squares of the Open Field (5.0 [3.0; 8.5]), which indicated a high level of anxiety in these animals [17]. In addition, after bilateral microinjection of glycine, there was a significant difference in the number of grooming patterns between Wistar and Sprague Dawley male rats (p=0.02) (Figure 4). However, subsequent microinjections of 20 μM strychnine led to a decrease in the number of grooming patterns in male Wistar rats (3.50 [1.75; 6.0]) compared with the number of grooming patterns during stimulation of inhibition in neural networks, and therefore the statistical significance of the differences between the number of grooming patterns in males of two stocks disappeared (see Figure 4).

Thus, bilateral administration of glycine/strychnine revealed inter-stock differences in the behavioral response to stimulation/inhibition of glycinergic transmission in the hypothalamic mPOA when passing the Open Field test.

## Discussion

In this study, the consummatory sexual behavior of intact animals significantly differed (p<0.05) between the Sprague Dawley and Wistar male rats. The most notable was the close-to-complete absence of the “mounting” pattern in male Wistar stocks. There is a report that male Wistar rats make fewer mountings and more intromissions than Sprague Dawley rats [4]. In addition, male Wistar rats needed more time and intromissions to achieve ejaculation. This difference may indicate a lower efficiency of copulation performance of male Wistar rats, which may be due to a higher level of anxiety in these specific stocks. According to the “funnel” model [18], anxious males need more time checking the safety of the environment, thereby reducing the efficiency of their copulation performance.

The open field test confirmed the higher level of anxiety in intact male Wistar stocks. The number of grooming patterns in these animals was higher than that in the Sprague Dawley stocks. Moreover, grooming pattern in Wistar rats was “incomplete” indicating a higher degree of anxiety [17, 19].

The revealed differences in sexual behavior between the male Sprague Dawley and Wistar stocks are probably due to the difference in the structure of inhibitory processes in the neural networks of the hypothalamic mPOA. Analysis of sexual behavior after bilateral microinjections of glycine and strychnine into the mPOA revealed differences between these two stocks. Microinjections of glycine into the mPOA of Wistar males resulted in their complete or partial inability to copulate, while the same microinjections in Sprague Dawley males, on the contrary, led to an increased copulation performance, as evidenced by a reduced ejaculation latency period [15].

It is known that glycine can interact not only with the glycine-specific receptor having an inhibitory effect on neurons [20] but also with the regulation site of the ionotropic glutamate receptor, which selectively binds N-methyl-D-aspartate (NMDA receptor) [21]. Therefore, considering that glycine microinjections led to the disappearance of copulation in male Wistar rats and, conversely, increased the efficiency of copulation performance in Sprague Dawley rats, we assume that animals of these two tocks have different ratios of glycine and NMDA receptors. This hypothesis is supported by the appearance of the “mounting” pattern in male Wistar stocks after microinjections of strychnine into the mPOA. Since the “mounting” is considered as an independent pattern during copulation [15], its occurrence can be seen as an enrichment of the male behavioral repertoire. In Sprague Dawley rats, microinjections of strychnine into the mPOA had no significant effect on the male behavior.

The involvement of the mPOA glycinergic neurotransmission in the regulation of sexual behavior in male Sprague Dawley and Wistar stocks may also be mediated through the regulation of anxiety. This assumption is confirmed by the results of the open field test during pharmacological modification of glycinergic neurotransmission in the mPOA. Microinjections of glycine increased the number of “incomplete” grooming patterns in male Wistar rats, while strychnine injections had a lesser effect.

Analysis of social interaction (Crowley test) among intact animals and animals after the activation/inhibition of glycinergic transmission revealed no difference between the Sprague Dawley and Wistar stocks; therefore, social activity is not a factor influencing the difference in the male sexual behavior of these two stocks.

Therefore, the statistically significant difference in the male sexual behavior between Sprague Dawley and Wistar rats found in the study may be due to different ratios of glycine/NMDA receptors in the hypothalamic mPOA and associated with different levels of anxiety in these two stocks. The latter factor probably reflects a difference between the glycinergic transmission mechanisms in the hypothalamic mPOA. These features should be taken into account when choosing a model rat stock for experiments with sexual behavior.

It is also of interest to study the genetic determinants of glycine mPOA receptors and reveal more details on the glycinergic mPOA transmission and its role in individual sexual behavior.

## Conclusion

To study the relationship between the level of anxiety and sexual behavior, the choice of Wistar rats is optimal since male sexual behavior in this stock is more sensitive to stress than that in Sprague Dawley rats. However, to model male sexual dysfunction not associated with anxiety, the use of Sprague Dawley male rats should be preferred as these animals show more stable sexual behavior, which is less dependent on the level of anxiety.
